# Thermal Index for early non-invasive assessment of brain injury in newborns treated with therapeutic hypothermia: preliminary report

**DOI:** 10.1038/s41598-021-92139-6

**Published:** 2021-06-15

**Authors:** W. Walas, A. Mączko, Z. Halaba, M. Bekiesińska-Figatowska, I. Miechowicz, D. Bandoła, Z. Ostrowski, M. Rojczyk, A. J. Nowak

**Affiliations:** 1 Paediatric and Neonatal Intensive Care Unit, Institute of Medical Sciences, University of Opole, Opole, Poland; 2 Department of Paediatrics, Institute of Medical Sciences, University of Opole, Opole, Poland; 3 Department of Diagnostic Imaging, Institute of Mother and Child, Warsaw, Poland; 4 Department of Computer Science and Statistics, Poznan University of Medical Sciences, Poznan, Poland; 5 Biomedical Engineering Laboratory, Department of Thermal Technology, Silesian University of Technology, Gliwice, Poland; 7 Paediatric and Neonatal Intensive Care Unit, University of Opole, Opole, Poland

**Keywords:** Neurophysiology, Physiology, Health care, Neurological disorders

## Abstract

Perinatal asphyxia (PA) is the 3rd most common cause of neonatal death and one of the most common causes of severe neurological impairments in children. Current tools and measurements mainly based on the analysis of clinical evaluation and laboratory and electrophysiological tests do not give consistent data allowing to predict the severity of hypoxic-ischemic encephalopathy (HIE) until a magnetic resonance imaging (MRI) score is performed. The aim of this work is to evaluate the usefulness of the new index, called Thermal Index (TI) in the assessment of the degree of brain damage in newborns in the course of therapeutic hypothermia (TH) due to PA. This was a prospective, observational, pilot study which did not require any changes in the applicable procedures. Analysis has been applied to six newborn babies treated with TH in Neonatal/Paediatric ICU in University Hospital in Opole in 2018 due to PA. They all met criteria for TH according to the current recommendations. Brain MRI was performed after the end of TH when the children were brought back to normal temperature, with the use of a 1.5 T scanner, using T1-, T2-weighted images, fluid-attenuated inversion recovery (FLAIR), inversion recovery (IR), susceptibility-weighted imaging (SWI), and diffusion-weighted imaging (DWI). The images were assessed using MRI score according to the scoring system proposed by Weeke et al. The Thermal Index assessing endogenous heat production was calculated according to the formula proposed in this paper. A high, statistically significant positive correlation was found between MRI scores and TI values (0.98; p = 0.0003) in the 1st hour of therapy. High correlation with MRI assessment, the non-invasiveness of measurements and the availability of results within the first few hours of treatment, allow authors to propose the Thermal Index as a tool for early evaluating of the brain injury in newborns treated with TH. Further research is required to confirm the usefulness of the proposed method.

## Introduction

Perinatal asphyxia (PA) represents the 3rd most common cause of neonatal death. This means that all over the world, almost 600,000 newborns die every year, and at least as many develop hypoxic-ischemic encephalopathy (HIE), which is one of the most common causes of severe neurological deficits in children, presently in approx. 15 out of 10,000 live births^1,2^. Among the various methods tested for efficacy in newborns with PA, therapeutic hypothermia (TH) has proved to be useful in clinical practice^3,4^. Early evaluation of the degree of brain damage in newborns after PA is of great importance in determining the appropriate management and prognosis. In the initial phase of treatment this assessment is based on a complex analysis of different factors, such as clinical evaluation of neurological status, laboratory, and electrophysiological tests. Although hypoxia markers and electrophysiological tests have been studied many times and are quite widely used in clinical practice, there are currently no consistent data to establish thresholds corresponding to HIE severity and the predictive value of individual tests for subsequent outcomes leaves a great deal to be desired^5,6^. Neuroimaging, especially magnetic resonance imaging (MRI), plays a special role in determining the prognosis of newborns after PA. Both conventional and newer MRI techniques are used and MRI is nowadays a standard tool for determining the pattern and severity of brain injury as well as the prognosis in infants with HIE^7–12^. Scales for objective, the numerical assessment of the degree of brain damage in neonates with HIE were developed^13–17^. However, while MRI is very useful for predicting the consequences of PA, it cannot be performed in an Intensive Care Unit (ICU), so it is not suitable for assessing neonatal brain damage during TH treatment. Therefore early assessing the degree of brain damage and prognosis in newborns with HIE treated with TH remains a challenge and new methods are being sought for.

Because PA causes a dysfunction of the thermoregulation centre, the methods of assessing the degree of brain injury and prognosis based on a non-invasive estimation of endogenous heat production have also garnered interest recently, and preliminary results are encouraging. The relationship between disturbances in thermoregulation and prognosis in patients with traumatic brain injury and after cardiac arrest has been proven^18–27^. Even some simple methods of assessing brain damage in adult patients after cardiac arrest and in newborns after PA have been proposed^28–30^. However, these methods result in a Heat Index (HI)^28^ and utilize the temperature of the cooling water only. Hence, from the heat transfer point of view, its theoretical basis is limited, because the temperature of the cooling water is not the only factor that defines the heat rate during TH.

A new methodology of analysing the cooling process (allowing to estimate thermogenesis) of newborns with HIE undergoing TH has been presented in^31,32^. The proposed method, with roots in heat transfer theory, takes into account more factors influencing the heat transfer and employs only the non-invasive thermal measurements. Following this methodology, a Thermal Index (TI) is now proposed in order to early assess the degree of brain damage in newborns after PA treated with TH. The aim of the presented pilot study is to evaluate the usefulness of this method in clinical practice. For this purpose, in newborns with PA treated with TH, we examined the correlation between TI and the MRI, which is an approved marker of HIE. We also examined the correlation between MRI and other popular markers of PA, such as Apgar score, pH, AspAt, AlAt, CK-MB and HSTNT to find out if TI is a better-suited marker of brain damage.

## Type of study

This was a prospective, observational pilot study that did not require any changes from the standard treatment of included patients.

## Materials and methods

### Patients

Six neonates with PA, treated using TH in Paediatric ICU (PICU) in Opole, Poland in 2018 were included in the study after obtaining the consent of their legal guardians. The newborns were transported to PICU from neonatal departments, where they were qualified for TH based on clinical and biochemical eligibility criteria. Inclusion criteria were in accordance with current recommendations:

1. gestational age at birth ≥ 35 weeks, and
2. birth weight ≥ 1800 g, and
3. Apgar score ≤ 5 in 1st, 3rd, 5th, and 10th minute after birth and/or the need of artificial ventilation for neurological reasons in the 10th minute after birth, and
4. deep acidosis: pH in the cord or arterial blood ≤ 7.0 and/or Base Excess (BE) ≤ 16 mmol/l in the cord, arterial or venous blood in the 1st hour after birth, and
5. neurological disorders: variable states of consciousness and at least one of the symptoms: muscle hypotension, abnormal reaction to stimuli (including abnormal oculomotor and/or pupillary reflex), no or weak sucking reflex, seizures.

Exclusion criteria were as follows:

1. > 6 h after birth, and/or
2. gestational age < 35 weeks, and/or
3. birth weight < 1800 g, and/or
4. severe birth defects with poor prognosis, and/or
5. severe coagulopathy, and/or
6. massive intracranial hemorrhage, and/or
7. extremely severe hypoxia with Apgar score = 0 at 10th minute after birth.

Immediately after admission to PICU, amplitude integrated electroencephalography (aiEEG) was performed as an additional criterion for qualifying for TH treatment in doubtful situations.

### Therapeutic hypothermia treatment

Newborns after PA admitted to Paediatric ICU were qualified for TH treatment based on clinical/biochemical criteria. Cranial ultrasound, laboratory tests and aiEEG were performed within 30 min of admission. After confirming the indications and ruling out contraindications, TH was started with the Olympic Cool-Cap (Olympic Medical, division of Natus, USA) device. The treatment was carried out for 72 h under rectal temperature control, which was maintained in the range of 34–35 °C. The rewarming rate was 0.2 °C/h. The treatment was conducted in accordance with the rules adopted in the ward and the study did not affect its course.

### Collecting data

#### Clinical data

Based on the documentation from the delivery room, the maternal pyrexia, prolonged rupture of membranes, type of delivery (natural/caesarean section), gender (male/female), gestational age (weeks), birth weight (grams), Apgar scores in the 1, 5, and 10 minutes after birth, arterial cord Base Excess, pH on first blood gas and resuscitation at birth were recorded. Based on the admission data and treatment documentation, the rectal temperature, type of respiration, inotropic support, heart rate, Sarnat score and aiEEG at admission as well as seizure onset age, age of start active cooling, timing of MRI and blood positive culture were recorded.

#### Laboratory tests

Within 30 min of admission to the department, selected biochemical parameters were evaluated: pH (arterial blood), Aspartate Aminotranspherase (AspAt), Alanine Aminotranspherase (AlAt), Creatine Kinase [Muscle, Brain] (CKMB), High-Sensitivity Troponin T (HSTNT).

#### MRI assessment

The MRI was performed after the end of TH, in the 6th or 7th day after birth. Brain MRI was performed with use of a 1.5 T scanner, using T1-, T2-weighted images, fluid-attenuated inversion recovery (FLAIR), inversion recovery (IR), susceptibility-weighted imaging (SWI) and diffusion-weighted imaging (DWI) sequences. The images were assessed using MRI score (MRIS) according to the scoring system proposed by Weeke et al.^16^. The higher score, the more serious brain injury. The lowest possible score was 0, and the highest was 55.

#### Thermal Index (TI)

As described by Walas et al.^32^ the heat balance for the neonate undergoing TH reads

1 $$ Q_{m}  = \frac{{{\text{d}}\,U}}{{{\text{d}}\,\tau }} + Q_{{skin}}  + Q_{{resp}} $$

where *Q*_*m*_ represents the rate of metabolic heat production, *Q*_*skin*_ is the rate of heat dissipated through the skin (to the local indoor environment) while *Q*_*resp*_ stands for the rate of heat exchanged due to respiration. These three heat rates are all expressed in watts (W). The time derivative on the right-hand side of the equation accounts for changes of neonate’s body internal energy *U* with respect to time. In the proposed study, internal energy *U* has been determined in a standard way as a function of tissues temperatures (based on two terms related to skin and core compartment temperature measurements)

2 $$ U = \left( {1 - \alpha _{{skin}} } \right)\,W\,c_{b} \,T_{{core}}  + \alpha _{{skin}} \,W\,c_{b} \,T_{{skin}} $$

where *W* denotes the body weight of the newborn and *c*_*b*_ stands for the specific heat of the tissue (usually *c*_*b*_ = 3490 J kg^−1^ K^−1^). Coefficient ${\alpha }_{skin}$ stands for the ratio of the rate of blood flowing to the skin. For the state of hypothermia, this parameter was determined as ${\alpha }_{skin}$ = 0.1721. The core temperature, as well as the temperature of the head skin and abdomen skin are measured using standard Cool Cap temperature sensors. It should be mentioned those temperatures are recorded by the Cool Cap system and can be further processed to calculate internal energy *U*.

Summation of *Q*_*skin*_ and *Q*_*resp*_ represents the total heat rate exchanged by the neonate with cooling water and a local indoor environment. In case of the selective hypothermia, the dominant term of *Q*_*skin*_ heat rate is the heat exchanged between the neonate’s head and cooling water flowing through a cooling cap. The amount of heat exchanged in this way in the early phase of TH (in our study this is during the first hour) can be treated as a driving quantity of TH process.

This amount of heat, marked here as *Q*_*cooling*_, is uniquely defined by the inlet and outlet temperatures of the cooling water and its volume flow rate. Those temperatures are again measured at every minute and recorded by the Cooling Cap system. However, the volume flow rate has to be measured separately. An appropriate water flow meter has been designed and connected to the cooling device through standard inlet/outlet ports. Finally

3 $$ Q_{{cooling}}  = c_{w} \,\rho _{w} \sum\limits_{{i = 1}}^{{60}} {Q_{{w,i}} } \,\Delta T_{{w,i}} $$

where *Q*_*w,i*_ is the volume flow rate of the cooling water measured at every *i*th minute of the first hour and ∆*T*_*w,i*_ stands for a mean temperature increase of the cooling water at this time. The water specific heat and water density are marked by *c*_*w*_ (4186.8 J kg^−1^ K^−1^) and ρ_*w*_ (1000 kg m^−3^), respectively.

It should be stressed that the decrease of neonate’s core temperature, or more precisely decrease of internal energy ∆*U* during the same time period (i.e. 1 h for present study) can be seen as a direct effect of the cooling process. The ratio of these two quantities, i.e. ∆*U* calculated according to Eq. (2) and *Q*_*cooling*_ determined according to Eq. (3), represents the effectiveness of the cooling process, which depends on the degree of brain damage. Finally, the above described ratio TI is related to neonate’s body weight *W* and scaled to make results comparable with the MRIS^16^ results:

4 $$ TI = \frac{1}{W}\frac{{\Delta U}}{{Q_{{cooling}} }} = \frac{1}{W}\frac{{U_{{initial}}  - U_{{after\;1\;hour}} }}{{Q_{{cooling}} }} $$

More details can be found in the study by Walas et al.^32^.

### Statistical analysis

Since the MRIS is measured on an ordinal scale, nonparametric tests were used. In order to investigate the relationship between the MRIS and other analyzed parameters, the Spearman rank correlation coefficient was calculated. Kendall's W coefficient of concordance with chi-square test was calculated to determine the agreement between the MRIS and the TI. First, however, the min–max normalization was used for MRIS, TI and pH to change their values to the range [0; 1]. Statistica 12 by StatSoft and PQStat by PQStat Software were used.

### Ethics statement

The research is not a clinical trial and therefore does not need to be registered. The study protocol was consistent with the ethical guidelines of the Declaration of Helsinki and its later amendments and current EU guidelines and regulations. The study was approved by the Bioethics Committee for Research Studies at the Opole Medical Chamber (Approval No 271/2018 and 272/2018). Written informed consent was obtained from all legal guardians of all study participants.

## Results

**Figure 1:**
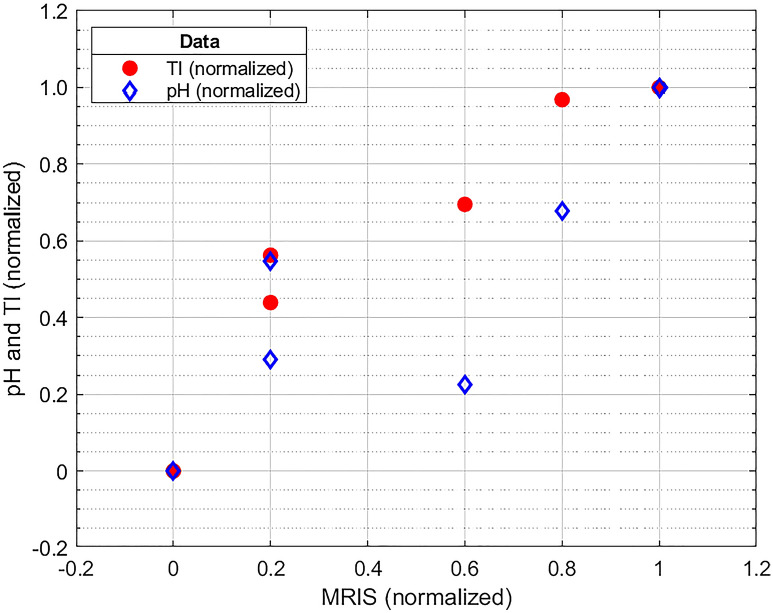
Correlation between the MRIS and the pH and TI scales (all variables were min–max normalized to [0; 1]).

We included 6 newborns in the study. All newborns met the clinical and biochemical eligibility criteria for TH prior to admission to the ICU. All of them had aiEEG immediately after admission, but there was no need to include this parameter in the TH decision. The characteristics of the group—data on delivery, condition of the newborn after birth and on admission to PICU, as well as the course of treatment is presented in Table 1. There were no maternal pyrexia or prolonged rupture of the membranes in any of the cases. None of the newborns presented seizures, none needed inotropic support at admission and none had positive blood culture at admission and during treatment. The levels of analysed parameters are presented in Table 2. Statistical analysis is presented in Table 3. and demonstrated in Fig. 1.

**Table 1:** Characteristics of the studied group.

Parameter	Patient					
	1 8.04.2017 23:20	2 2.07.2017 22:06	3 07.02.2018 17:58	4 22.02 14:18	5 29.07.2018 21:20	6 17.10.2018 14:05
**Characteristics of the studied group**						
Sex (m/f)	m	m	m	f	f	m
Type of delivery (n/cs)	n	cs	n	cs	n	cs
Gestational age (weeks + days)	41 + 0	40 + 1	39 + 0	40 + 2	40 + 0	39 + 4
Birth weight (g)	3290	2140	3200	3130	3260	3680
Resuscitation at birth	Inflation breaths	Intubation, mechanical ventilation	Inflation breaths	Intubation, mechanical ventilation	Inflation breaths	Inflation breaths
Arterial cord BE (mmol/L)	− 21.2	− 24.4	− 13.0	− 18.7	− 16.6	− 15.0
pH in 1th blood gas	6.81	6.72	7.01	7.02	6.89	7.04
Rectal temp at admission (^o^C)	33.5	33.1	34.2	34.6	36.0 ?	35.3
Respiration at admission	nCPAP	Mechanical ventilation	IF (BP)	Mechanical ventilation	Spontaneous breath	IF (NCPAP)
Heart rate at admission (bpm)	134	120	135	117	141	123
Sarnat score	Stage II	Not available (sedation)	Stage II	Not available (sedation)	Stage II	Stage II
aiEEG at admission	Moderately abnormal	Moderately abnormal	Moderately abnormal	Severely abnormal	Moderately abnormal	Moderately abnormal
Age of start of active cooling	3 h 38 min	3 h 2 min	3 h 34 min	3 h 46 min	2 h 53 min	3 h 37 min
Timing of MRI (day after birth)	6th	6th	7th	7th	7th	7th

*m* male, *f* female, *n* natural, *cs* caesarean section, *bpm* beats per minute, *IF (BP)* Infant Flow BiPhasic.

**Table 2:** Levels of analysed parameters in patients 1–6.

Parameter	Patient					
	Levels of analysed parameters					
Apgar 1. min	4	1	3	1	1	1
Apgar 5. min	7	3	5	4	5	8
Apgar 10. min	7*	4	6*	6*	6*	8*
pH	6.92	6.83	7.00	7.14	6.90	7.04
AspAt (U/l)	98	21	49	43	95	47
AlAt (U/l)	21	5	22	20	28	23
CK-MB (ng/ml)	63.89	81.75	37.40	49.15	140.40	20.60
HSTNT (ng/l)	617.60	76.43	91.36	83.13	151.10	141.90
TI	0.01891	0.00750	0.02212	0.03348	0.02556	0.03265
Deep grey matter*	0	0	0	0	0	0
White matter cortex*	0	0	0	4	3	4
Cerebellum*	0	0	0	0	0	0
Additional*	1	0	1	1	0	0
MRIS	1	0	1	5	3	4

*Artificial ventilation for neurological reasons needed in the 10th minute after birth * Weeke scale, *MRIS* MRI Score.

**Table 3:** Correlation between the MRI scale (MRIS) and the analyzed parameters (the Spearman rank correlation coefficient).

	Parameter	Rs coefficient	p-value
MRIS &	TI	0.9856	0.0003*
	Apgar 1. min	− 0.4116	0.4175
	Apgar 5. min	0.2794	0.5918
	Apgar 10. min	0.4620	0.3563
	pH	0.8117	0.0499*
	AspAt	− 0.0290	0.9565
	AlAt	0.3479	0.4993
	Ck-MB	− 0.3769	0.4615
	HSTNT	0.1160	0.8268

*Significant correlation (p < 0.05).

## Discussion

MRI is nowadays a standard tool for determining the pattern and severity of brain injury as well as the prognosis in infants with HIE. The usefulness of conventional and modern MRI techniques, such as: T2*-weighted imaging (GRE/T2*WI), susceptibility-weighted imaging (SWI), diffusion-weighted imaging (DWI), as well as the magnetic resonance spectroscopy (MRS), diffusion tensor imaging (DTI) has been proven^7–12^. Scoring scales have also been developed to quantify hypoxic brain injury^13–16^. Since MRI is considered to be the best predictor of brain damage, we looked for a correlation between it and Apgar score and biochemical test results. We used the scale proposed by Weeke et al.^17^. In our opinion, this scale is the most suitable for assessing brain damage in the first days of life because it covers the largest range of possible pathological changes and the broadest range of MRI techniques thus allowing for the most detailed assessment. Moreover, it offers a simpler and more unequivocal scoring method than other scales. The predictive value of the scoring system by Weeke et al. for outcome at 2 years of age and at school age has been proven^17^.

In our study, we found a significant positive correlation between the MRIS and TI (p = 0.0003). To determine whether TI is a better-suited predictor of brain damage than other commonly used indicators, we also examined the correlation between MRIS and Apgar score, pH, AspAt, AlAt, CK-MB and HSTNT. We have found also a noticeable positive correlation between MRIS and pH (p = 0.0499), but the strength of this correlation was weaker than for TI (Rs = 0.8117 vs. Rs = 0.9856). The compatibility between the MRIS and the TI was very high (Kendall’s W = 0.9928, the average Spearman rank correlation coefficient = 0.9855), although unfortunately, due to the very small sample size, it was not statistically significant (p = 0.0773). There was no significant correlation between the MRIS and any other considered parameters.

Our results are in line with those obtained by adult patients after cardiac arrest (CA). Murnin et al. proposed the use of estimated endogenous heat production to establish prognosis in patients after CA treated by TH. They estimated the endogenous heat production using the so-called Heat Index (HI), which was calculated based only on the temperature of water pumped by the cooling device. They found that in cardiac arrest patients receiving TH, greater heat generation is associated with better baseline health, reduced ischemic injury, and improved neurologic function, which results in higher metabolism^28^. These observations were confirmed by Uber et al., who also used HI. They demonstrated that increased energy required by a cooling device to cool a patient after CA to target temperature is associated with improved outcomes at hospital discharge^29^. Similar observations were made for neonates after PA treated by TH. Mietzsch et al. noted that the temperature of the active cooling medium correlates with the severity of brain injury diagnosed by MRI. A significantly higher cooling device output temperature was seen in infants with an unfavourable outcome. Infants with significant grey matter injury on MRI require less active cooling to maintain target temperature during TH. The authors concluded that the cooling device output temperature has the potential to be an easily accessible physiological biomarker and a predictor of injury and mortality in neonates with moderate or severe HIE^30^.

In our study, we used the methodology of the heat balance proposed by Bandoła et al.^31^, further developed by Walas et al.^32^. These authors presented examples of the measurements of the proposed parameters during TH in newborns, but they did not present the results of studies confirming the clinical usefulness of the proposed method. This methodology is based on a larger number of non-invasive measurements performed during TH treatment than used by Murnin et al., Uber et al. and Mietzsch et al.^28–30^. Therefore, it seems that it may be less susceptible to external factors while maintaining the value of full non-invasiveness. Unfortunately, the direct comparison of our results with the research results of the authors using HI is doubtful due to different cooling methods (Cool-Cap vs Whole Body Hypothermia) and collecting measurement data in a completely different stage of the therapy (the first hour of therapy vs minimum 2 h once patient's and cooling fluid temperatures are stable).

We found also a significant correlation between MRIS and pH, although the strength of this correlation was weaker than between MRIS and TI. This is consistent with other reports. Among the laboratory tests, the acid–base balance holds a special place, because metabolic acidosis is the second prequalification criterion for TH treatment. Shah et al., Ambalavanan et al. and Wayock et al. have demonstrated the importance of metabolic acidosis in predicting neonatal outcomes^33–35^. According to a systematic literature review and meta-analysis by Malin et al., a strong, consistent, and temporal association between low umbilical arterial pH and clinically important neonatal outcomes has been proven^36^.

In our study, we found no correlation between MRIS and the Apgar score in 1st, 5th and 10th minute after birth. This observation is in line with the literature^37,38^. We also found no correlation between MRIS and AspAt, AlAt, CKMB, HSTNT levels. Liver enzymes and myocardial injury biomarkers are often measured in neonates after PA, but their prognostic significance is ambiguous^39–45^.

## Limitations

Our pilot study was performed on a very small group of patients. We used selective head hypothermia in our patients, which makes it difficult to compare the results with the results of authors using whole body hypothermia. Another limitation is the lack of long-term follow-up. Therefore the conclusions must be treated with some reserve. However, we decided to publish our results because this is the first study to initially confirm the usefulness of a new non-invasive method for an early prognosis of HIE severity.

## Conclusion

Preliminary results allow us to propose the Thermal Index as a new non-invasive tool for early evaluation degree of brain injury in newborns undergoing TH, that shows significant correlation with the MRI assessment. It is necessary to confirm the obtained preliminary results on a larger group of patients.

## Data Availability

All calculations related to statistical analysis have been made in Statistica 12 by StatSoft and PQStat by PQStat Software. Thermal calculations and presentation of results have been prepared using Matlab.

## References

[1] Graham EM, Ruis KA, Hartman AL, Northington FJ, Fox HE (2008). A systematic review of the role of intrapartum hypoxia–ischemia in the causation of neonatal encephalopathy. Am. J. Obstet. Gynecol..

[2] World Health Organization. *Global Health Observatory (GHO)* (WHO, 2016). http://www.childmortality.org/. Accessed 10 April 2020.

[3] Jacobs SE, Berg M, Hunt R, Tarnow-Mordi WO, Inder TE, Davis PG (2013). Cooling for newborns with hypoxic–ischaemic encephalopathy. Cochrane Syst Rev..

[4] Wyllie J, Bruinenberg J, Roehr CC, Rüdiger M, Trevisanuto D, Urlesberger B (2015). European Resuscitation Council Guidelines for Resuscitation 2015 Section 7 Resuscitation and support of transition of babies at birth. Resuscitation.

[5] Liu W, Yang Q, Wei H, Dong W, Fan Y, Hua Z (2020). Prognostic value of clinical tests in neonates with hypoxic-ischemic encephalopathy treated with therapeutic hypothermia: A systematic review and meta-analysis. Front. Neurol..

[6] Walas W, Wilińska M, Bekiesińska-Figatowska M, Halaba Z, Śmigiel R (2020). Methods for assessing the severity of perinatal asphyxia and early prognostic tools in neonates with hypoxic-ischemic encephalopathy treated with therapeutic hypothermia. Adv. Clin. Exp. Med..

[7] Thayyil S, Chandrasekaran M, Taylor A, Bainbridge A, Cady EB, Chong WK, Murad S, Omar RZ, Robertson NJ (2010). Cerebral magnetic resonance biomarkers in neonatal encephalopathy: A meta-analysis. Pediatrics.

[8] Cheong JL, Coleman L, Hunt RW, Lee KJ, Doyle LW, Inder TE (2015). Prognostic utility of magnetic resonance imaging in neonatal hypoxic-ischemic encephalopathy: Substudy of a randomized trial. Arch. Pediatr. Adolesc. Med..

[9] Bekiesinska-Figatowska M, Duczkowska A, Szkudlinska-Pawlak S, Duczkowski M, Madzik J, Cabaj A, Krupa K, Peczkowski P, Bragoszewska H (2017). Diffusion restriction in the corticospinal tracts and the corpus callosum in neonates after cerebral insult. Brain Dev..

[10] Rana L, Sood D, Chauhan R, Shukla R, Gurnal P, Nautiyal H, Tomar M (2018). MR Imaging of hypoxic ischemic encephalopathy: Distribution Patterns and ADC value correlations. Eur. J. Radiol. Open..

[11] Mitra S, Kendall GS, Bainbridge A, Sokolska M, Dinan M, Uria-Avellanal C, Price D, Mckinnon K, Gunny R, Huertas-Ceballos A, Golay X, Robertson NJ (2019). Proton magnetic resonance spectroscopy lactate/N-acetylaspartate within 2 weeks of birth accurately predicts 2-year motor, cognitive and language outcomes in neonatal encephalopathy after therapeutic hypothermia. Arch. Dis. Child Fetal. Neonatal Ed..

[12] Lemmon ME, Wagner MW, Bosemani T, Carson KA, Northington FJ, Huisman TAGM, Poretti A (2017). Diffusion tensor imaging detects occult cerebellar injury in severe neonatal hypoxic-ischemic encephalopathy. Dev. Neurosci..

[13] Trivedi SB, Vesoulis ZA, Rao R, Liao SM, Shimony JS, McKinstry RC, Mathur AM (2017). A validated clinical MRI injury scoring system in neonatal hypoxic-ischemic encephalopathy. Pediatr. Radiol..

[14] Rutherford M, Ramenghi LA, Edwards AD, Brocklehurst P, Halliday H, Levene M (2010). Assessment of brain tissue injury after moderate hypothermia in neonates with hypoxic-ischaemic encephalopathy: A nested substudy of a randomised controlled trial. Lancet Neurol..

[15] Shankaran S, Barnes PD, Hintz SR, Laptook AR, Zaterka-Baxter KM, McDonald SA (2012). Brain injury following trial of hypothermia for neonatal hypoxic–ischaemic encephalopathy. Arch. Dis. Child Fetal Neonatal. Ed..

[16] Barkovich AJ, Hajnal BL, Vigneron D, Sola A, Partridge JC, Allen F (1998). Prediction of neuromotor outcome in perinatal asphyxia: Evaluation of MR scoring systems. AJNR Am. J. Neuroradiol..

[17] Weeke LC, Groenendaal F, Mudigonda K (2018). A novel magnetic resonance imaging score predicts neurodevelopmental outcome after perinatal asphyxia and therapeutic hypothermia. J. Pediatr..

[18] Haugk M, Testori C, Sterz F, Uranitsch M, Holzer M, Behringer W, Herkner H, Time to Target Temperature Study Group (2011). Relationship between time to target temperature and outcome in patients treated with therapeutic hypothermia after cardiac arrest. Crit. Care..

[19] Perman SM, Ellenberg JH, Grossestreuer AV, Gaieski DF, Leary M, Abella BS, Carr BG (2015). Shorter time to target temperature is associated with poor neurologic outcome in post-arrest patients treated with targeted temperature management. Resuscitation.

[20] Lyon RM, Richardson SE, Hay AW, Andrews PJ, Robertson CE, Clegg GR (2010). Esophageal temperature after out-of-hospital cardiac arrest: An observational study. Resuscitation.

[21] den Hartog AW, de Pont AC, Robillard LB, Binnekade JM, Schultz MJ, Horn J (2010). Spontaneous hypothermia on intensive care unit admission is a predictor of unfavorable neurological outcome in patients after resuscitation: An observational cohort study. Crit. Care..

[22] Lin S, Scales DC, Dorian P, Kiss A, Common MR, Brooks SC, Goodman SG, Salciccioli JD, Morrison LJ (2014). Targeted temperature management processes and outcomes after out-of-hospital cardiac arrest: An observational cohort study. Crit. Care Med..

[23] Hovdenes J, Røysland K, Nielsen N, Kjaergaard J, Wanscher M, Hassager C, Wetterslev J, Cronberg T, Erlinge D, Friberg H, Gasche Y, Horn J, Kuiper M, Pellis T, Stammet P, Wise MP, Åneman A, Bugge JF (2016). A low body temperature on arrival at hospital following out-of-hospital-cardiac-arrest is associated with increased mortality in the TTM-study. Resuscitation.

[24] Benz-Woerner J, Delodder F, Benz R, Cueni-Villoz N, Feihl F, Rossetti AO, Liaudet L, Oddo M (2012). Body temperature regulation and outcome after cardiac arrest and therapeutic hypothermia. Resuscitation.

[25] Wang HE, Callaway CW, Peitzman AB, Tisherman SA (2005). Admission hypothermia and outcome after major trauma. Crit. Care Med..

[26] Hsieh TM, Kuo PJ, Hsu SY, Chien PC, Hsieh HY, Hsieh CH (2018). Effect of hypothermia in the emergency department on the outcome of trauma patients: A cross-sectional analysis. Int. J. Environ. Res. Public Health..

[27] Erkens R, Wernly B, Masyuk M, Muessig JM, Franz M, Schulze PC, Lichtenauer M, Kelm M, Jung C (2020). Admission body temperature in critically ill patients as an independent risk predictor for overall outcome. Med. Princ. Pract..

[28] Murnin MR, Sonder P, Janssens GN, Henry CL, Polderman KH, Rittenberger JC, Dezfulian C, Post Cardiac Arrest Service (2014). Determinants of heat generation in patients treated with therapeutic hypothermia following cardiac arrest. J. Am. Heart Assoc..

[29] Uber AJ, Perman SM, Cocchi MN, Patel PV, Ganley SE, Portmann JM, Donnino MW, Grossestreuer AV (2018). Increased heat generation in postcardiac arrest patients during targeted temperature management is associated with better outcomes. Crit. Care Med..

[30] Mietzsch U, Radhakrishnan R, Boyle FA, Juul S, Wood TR (2020). Active cooling emperature required to achieve therapeutic hypothermia correlates with short-term outcome in neonatal hypoxic–ischaemic encephalopathy. J. Physiol..

[31] Bandoła D, Rojczyk M, Ostrowski Z (2018). Expermental setup and measurements of the heat transfer rate during newborn brain cooling process. Arch. Thermodyn..

[32] Walas W, Bandoła D, Ostrowski Z (2020). Theoretical basis for the use of non-invasive thermal measurements to assess the brain injury in newborns undergoing therapeutic hypothermia. Sci. Rep..

[33] Shah P, Beyene J, To T, Ohlsson A, Perlman M (2006). Postasphyxial hypoxic-ischemic encephalopathy in neonates: Outcome prediction rule within 4 hours of birth. Arch. Pediatr. Adolesc. Med..

[34] Ambalavanan N, Carlo WA, Shankaran S (2006). Predicting outcomes of neonates diagnosed with hypoxemic-ischemic encephalopathy. Pediatrics.

[35] Wayock CP, Meserole RL, Saria S, Jennings JM, Huisman TA, Northington FJ, Graham EM (2014). Perinatal risk factors for severe injury in neonates treated with whole-body hypothermia for encephalopathy. Am. J. Obstet. Gynecol..

[36] Malin GL, Morris RK, Khan KS (2010). Strength of association between umbilical cord pH and perinatal and long term outcomes: Systematic review and meta-analysis. BMJ.

[37] Laptook AR, Shankaran S, Ambalavanan N, Carlo WA, McDonald SA, Higgins RD, Das A, Hypothermia Subcommittee of the NICHD Neonatal Research Network (2009). Outcome of term infants using apgar scores at 10 minutes following hypoxic-ischemic encephalopathy. Pediatrics.

[38] Shah P, Anvekar A, McMichael J, Rao S (2015). Outcomes of infants with Apgar score of zero at 10 min: the West Australian experience. Arch. Dis Child Fetal Neonatal Ed..

[39] Muniraman H, Gardner D, Skinner J, Paweletz A, Vayalakkad A, Chee YH, Clifford C, Sanka S, Venkatesh V, Curley A, Victor S, Turner MA, Clarke P (2017). Biomarkers of hepatic injury and function in neonatal hypoxic ischemic encephalopathy and with therapeutic hypothermia. Eur J Pediatr..

[40] Agrawal J, Shah GS, Poudel P, Baral N, Agrawal A, Mishra OP (2012). Electrocardiographic and enzymatic correlations with outcome in neonates with hypoxic-ischemic encephalopathy. Ital. J. Pediatr..

[41] Türker G, Babaoğlu K, Gökalp AS, Sarper N, Zengin E, Arisoy AE (2004). Cord blood cardiac troponin I as an early predictor of short-term outcome in perinatal hypoxia. Biol. Neonate..

[42] Montaldo P, Rosso R, Chello G, Giliberti P (2014). Cardiac troponin I concentrations as a marker of neurodevelopmental outcome at 18 months in newborns with perinatal asphyxia. J. Perinatol..

[43] Shastri AT, Samarasekara S, Muniraman H, Clarke P (2012). Cardiac troponin I concentrations in neonates with hypoxic-ischaemic encephalopathy. Acta Paediatr..

[44] Trevisanuto D, Picco G, Golin R, Doglioni N, Altinier S, Zaninotto M, Zanardo V (2006). Cardiac troponin I in asphyxiated neonates. Biol. Neonate..

[45] Zhou WJ, Yu F, Shi J, Yang H, Zou SJ, Jiang YM (2016). Serum levels of cardiac troponin I in asphyxiated neonates predict mortality. Clin. Lab..

